# Serial analysis of gene expression (SAGE) in normal human trabecular meshwork

**Published:** 2011-04-08

**Authors:** Yutao Liu, Drew Munro, David Layfield, Andrew Dellinger, Jeffrey Walter, Katherine Peterson, Catherine Bowes Rickman, R. Rand Allingham, Michael A. Hauser

**Affiliations:** 1 Center for Human Genetics, Duke University Medical Center, Durham, NC; 2 Department of Ophthalmology, Duke University Medical Center, Durham, NC; 3 Department of Cell Biology, Duke University Medical Center, Durham, NC; 4 Section on Molecular Structure and Functional Genomics, National Eye Institute, National Institutes of Health, Bethesda, MD

## Abstract

**Purpose:**

To identify the genes expressed in normal human trabecular meshwork tissue, a tissue critical to the pathogenesis of glaucoma.

**Methods:**

Total RNA was extracted from human trabecular meshwork (HTM) harvested from 3 different donors. Extracted RNA was used to synthesize individual SAGE (serial analysis of gene expression) libraries using the I-SAGE Long kit from Invitrogen. Libraries were analyzed using SAGE 2000 software to extract the 17 base pair sequence tags. The extracted sequence tags were mapped to the genome using SAGE Genie map.

**Results:**

A total of 298,834 SAGE tags were identified from all HTM libraries (96,842, 88,126, and 113,866 tags, respectively). Collectively, there were 107,325 unique tags. There were 10,329 unique tags with a minimum of 2 counts from a single library. These tags were mapped to known unique Unigene clusters. Approximately 29% of the tags (orphan tags) did not map to a known Unigene cluster. Thirteen percent of the tags mapped to at least 2 Unigene clusters. Sequence tags from many glaucoma-related genes, including myocilin, optineurin, and WD repeat domain 36, were identified.

**Conclusions:**

This is the first time SAGE analysis has been used to characterize the gene expression profile in normal HTM. SAGE analysis provides an unbiased sampling of gene expression of the target tissue. These data will provide new and valuable information to improve understanding of the biology of human aqueous outflow.

## Introduction

Primary open-angle glaucoma (POAG, OMIM 137760) is the most common form of glaucoma, which is the leading cause of irreversible vision loss worldwide [1]. POAG is characterized by progressive loss of retinal ganglion cells and visual field in the absence of a known secondary cause. Well recognized risk factors for the development of POAG are elevated intraocular pressure (IOP), positive family history of glaucoma, refractive error, and African ancestry [2,3]. As a complex genetic disorder, there is a strong hereditary component to POAG; first-degree relatives of affected individuals have a 7–10 fold higher risk of developing POAG than the general population [4-6]. Several regions in the human genome have been linked to POAG [2]. To date, several genes including myocilin (*MYOC*), optineurin (*OPTN*), WD repeat domain 36 (*WDR36*), and cytochrome P450, family 1, subfamily B, polypeptide 1 (*CYP1B1*) have been implicated in POAG, but mutations in these genes account for less than 10% of POAG cases [7-10].

Linkage analyses are useful in determining regions of interest for complex diseases. However, linkage regions often contain dozens or even hundreds of genes. Although it is possible to sequence all genes within a linked locus using high-throughput second-generation sequencing, it is important to prioritize any identified sequence changes for further follow-up. Prioritizing genes for further analysis requires the use of other methods to provide complementary information, in an approach which we have termed genomic convergence [11]. This approach combines multiple forms of genome-wide data such as linkage, gene expression analysis and association studies to identify and prioritize candidate susceptibility genes for complex disorders [11,12]. Genome-wide association studies have been widely used to identify the risk factors for POAG and exfoliation glaucoma [13-15] but generate a very large number of candidate susceptibility genes. Gene expression data from ocular tissues will help in the interpretation and prioritization of this large number of candidate genes.

Expression profiling is commonly performed by either microarray or serial analysis of gene expression (SAGE) [16,17]. SAGE involves direct measurement of mRNA transcripts and generates a non-biased gene expression profile without regard to selection of a reference sample [16,18]. Advantages of SAGE include the power to identify fine variations in expression levels and the ability to detect novel transcripts without prior knowledge of gene sequence. It thus provides unique advantages over the traditional microarray-based approach for expression studies. In contrast, microarray gene expression profiling is based on the use of pre-designed probes for selected genes, or genome annotation [19]. Microarray analysis then measures the level of gene expression relative to a reference sample (e.g., tissue of a different type, or from a different individual) [17,19,20].

Non-SAGE expression analyses have been reported with human trabecular meshwork (HTM) and/or cultured HTM cells. The first analysis of gene expression in the trabecular meshwork was performed in 1990: Tripathi and coworkers examined levels of HLA expression in HTM [21]. Gonzales and coworkers [22] performed the first genome-wide expression analysis a decade later. They constructed a PCR-amplified cDNA library containing 1,060 clones from a non-glaucomatous HTM. Several genome-wide analyses have subsequently expanded our knowledge of gene expression in HTM [23-32]. To date, most studies have used a microarray-based approach with primary or cultured HTM cells. We report here the analysis of HTM obtained from three individuals using Long SAGE (using 17 base pair sequence tags) [33]. The present work aims to further our understanding of gene expression in the HTM, in support of an eventual understanding of the pathophysiology underlying determinants of ocular outflow facility.

## Methods

### Procurement of tissue and RNA extraction

Donor human eyes were obtained from the North Carolina Eye Bank (NCEB, Winston-Salem, NC). Immediately after enucleation, donated eyes were incised through the pars plana, the globe was immersed in RNALater (Ambion, Austin, TX), and was placed in storage at 4 °C. Within 24 h of death the trabecular meshwork (TM) was dissected using an operating microscope and stored at −80 °C until RNA isolation. De-identified clinical information and medical records were reviewed. There was no history of glaucoma, steroid use, or ocular trauma. Details regarding the donors and donor eyes are listed in Table 1. Medical record review and dissection of the TM was performed by a glaucoma trained subspecialist (R.R.A.).

**Table 1:** Human Donor eyes used for SAGE libraries.

**Sample ID**	**Age**	**Race**	**Gender**	**PMI (h)**	**Ocular history**	**PCD**	**Notes**
201	25	Eur	F	1.08	No	Anoxic brain injury	Anorexia, bulimia, depression, heart murmur
625	42	Eur	F	7.98	Yes	Pneumonia	Proliferative DR
784	68	Eur	M	2.92	No	Lung cancer	Prostatectomy, COPD, HTN, OA

Eur: European descent; F: female; M: male; PMI: post mortem interval; PCD: Primary cause of death; DR: diabetic retinopathy; COPD: chronic obstructive pulmonary disease; HTN: hypertension; OA: osteoarthritis.

Total RNA was extracted from the TM of one eye per donor using TRIzol (Invitrogen, Carlsbad, CA) followed by isopropanol precipitation. RNA quality was assessed by visualization in denaturing agarose gel electrophoresis and the 260 nm/280 nm ratio of absorbance. RNA concentration was calculated according to the absorbance measurement at 260 nm.

### Synthesis and analysis of SAGE libraries

Individual SAGE libraries from the 3 HTM samples were constructed with 5 µg RNA using the I-SAGE Long kit from Invitrogen. NlaIII was used as the anchoring enzyme. Standard methodologies were used according to the manufacturer’s recommendations [34]. SAGE libraries were sequenced at Agencourt Bioscience (Beverly, MA).

The SAGE 2000 software version 4.5 was used to extract and tabulate SAGE tags (17 base pairs in length) for each library. SAGE tags that matched to multiple genomic locations were removed. To minimize the background noise and false-positive results, only unique tags with a minimum of 2 counts in at least one of the three libraries were used for a gene match. The best gene match for each reliable tag was assigned using resources available at the Cancer Genome Anatomy Project (CGAP) SAGE Genie website [35] with the recent version of SAGE Genie library file (released November, 2009). Specifically, SAGE Genie’s “Best gene for the tag” table was used to match each long tag to its best Unigene cluster match. In most cases, a non-redundant assignment was made. Unigene clusters were mapped to the human genome assembly. Tag sequences, tag counts, and gene associations were stored in a relational database for subsequent analysis using Microsoft Access software (Redmond, WA). All SAGE data collected through this project has been has been deposited in NEIBank [36]. This expression data is freely available to researchers.

## Results

### SAGE libraries

Three SAGE libraries were produced, one from each donor, according to the standard protocol. Donor eyes were obtained within 1, 3, or 8 h postmortem from Caucasian donors of European descent that ranged in age from 25 to 68 years (Table 1). One individual (sample 625) had a history of proliferative diabetic retinopathy. None had any history of glaucoma, steroid use, or elevated intraocular pressure.

A total of 298,834 total tags were extracted from the SAGE libraries. Characteristics of the tags found in the three SAGE libraries are shown in Table 2. There were 107,325 unique tags collectively in the three separate libraries. Each library contained approximately 6,000 mapped unique Unigene clusters. Altogether, 10,329 unique Unigene clusters were mapped. After excluding singleton tags, the proportion of unmapped (orphan) tags ranged from 21% to 26%, which is comparable to the 20%–30% reported from other SAGE libraries [12,37,38]. Unique tags mapping to more than 2 Unigene clusters were removed from further analysis. Library 784 was sequenced to a greater depth than the other libraries, and thus contained the largest number of unique tags.

**Table 2:** Summary of the three HTM SAGE libraries.

**Donor**	**Total tags**	Unique tags *	**Unique tag counts 1**	**Unique tag counts ≥2**	**Unique Unigene clusters**	**Redundant Unigene clusters‡**	**Orphan tags﻿§**
201	96842	37212	27350	9862	6830 (69%)	949 (10%)	2083 (21%)
625	88126	36092	27106	8986	6214 (69%)	911 (10%)	1861 (21%)
784	113866	58216	48660	9556	5200 (54%)	1895 (20%)	2461 (26%)
Total	298834	107325†	92702†	17993†	10329 (58%)†	2371 (13%)†	5293 (29%)†

*Unique tags: Number of different tag sequences occurring in each library; ‡ Redundant Unigene clusters: unique tags with at least 2 counts in one library that map to more than 2 loci in human genome (NCBI build 37); §Orphan tags: unique tags with at least 2 counts in one library that do not map to any known Unigene cluster; † The total number here is for all unique tags in all three libraries instead of the sum-up. The percentage was calculated based on the sum of unique tags with at least 2 counts.

**Figure 1:**
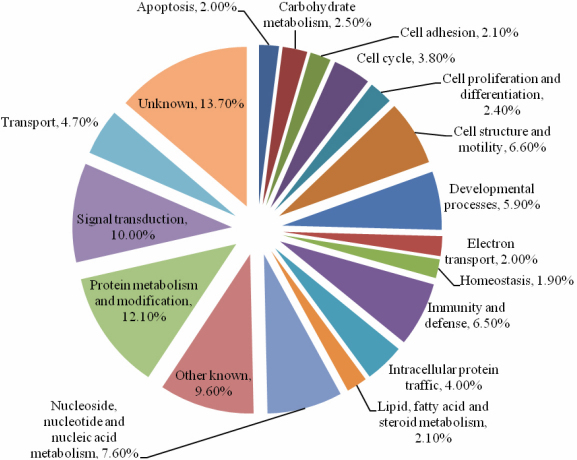
Functional categories based on the molecular process for top 650 genes with more than 30 tags in all three libraries. The gene ontology analysis was performed using PANTHER classification system (Protein ANalysis THrough Evolutionary Relationships).

The 650 genes that each comprise more than 0.01% of the total transcriptome (30 total tags or greater) were categorized by gene function using the PANTHER classification system (Protein ANalysis THrough Evolutionary Relationships) [39], as shown in Figure 1. The main functional categories included cell adhesion, cell structure and mobility, apoptosis, signal transduction, transport, and protein metabolism.

**Figure 2:**
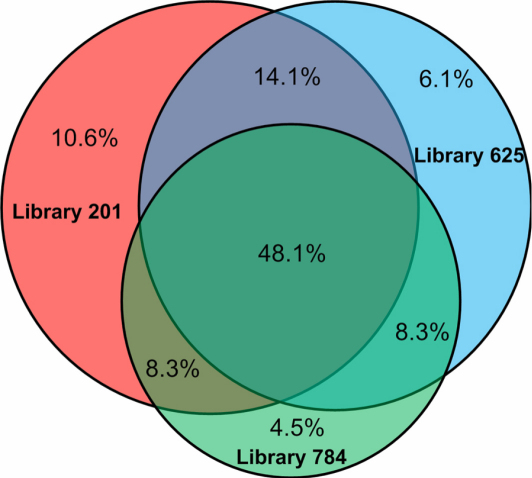
Venn’s diagram to compare the genes expressed in the three libraries #201, #625, and #784. All the percentage calculation was based on the total number of unique Unigene clusters combined from all three libraries.

We next examined genes that were expressed in multiple libraries: 56% were expressed in at least two libraries, while 48% were expressed in all three libraries (Figure 2). Expressed genes were mapped to known glaucoma loci, including GLC1B through GLC1D, GLC1F, and GLC1H through GLC1N. Appendix 1 lists only those genes that were found in all three libraries, while Appendix 2 lists those that were expressed in any single library.

The most abundantly expressed tags were those associated with components of ribosomal proteins. Because these house-keeping genes are commonly observed in SAGE libraries from various tissue types, they were removed from further analysis. The 40 remaining most highly expressed tags, with tag counts ranging from 200 to 3,511, are shown in Table 3. The most highly expressed non-ribosomal tag is an unnamed transcribed locus (UniGene Hs.703108). Two proteins considered to be HTM markers were represented by more than 120 tags in each library: *MGP* (matrix GLA protein) and *CHI3L1* (Chitinase 3-like 1) [40]. Three of the four genes reported to cause POAG, *MYOC*, *OPTN* and *CYP1B1*, were expressed in all three libraries, while *WDR36* was expressed in only one. Flotillin and gamma-synuclein, proteins which interact with myocilin, were expressed in all samples [41,42]. *Rab8* (ras-related protein Rab-8A) and *TBK1* (TANK-binding kinase 1), which interact with *OPTN,* were also expressed in all three libraries [2,43,44]. Sequence tags from 2 recently identified glaucoma-related genes, lysyl oxidase 1 (*LOXL1*; associated with exfoliation glaucoma), and caveolin 1 and caveolin 2 (associated with POAG) were expressed in at least two libraries [13,15]. The complete expression profiles can be found at Eyebrowse.

**Table 3:** Forty mapped genes with most highly expressed SAGE tags in the three HTM libraries (After the removal of ribosomal proteins).

**Tag**	**Tag counts (%†)**	**# of libraries**	**Unigene ID**	**Gene symbol**	**Gene description**	**Location**
TTCATACACCTATCCCC	3511 (1.17)	3	Hs.703108		Transcribed locus	
CCCTACCCTGTTACCTT	1594 (0.53)	3	Hs.522555	APOD	Apolipoprotein D	3q26.2-qter
CACCTAATTGGAAGCGC	1254 (0.42)	3	Hs.150324	LOC100133315	Similar to hCG1640299	11q13.4
TGATTTCACTTCCACTC	1143 (0.38)	3	Hs.634715		Transcribed locus	
GCCCCTGCTGACACGAG	1090 (0.36)	3	Hs.433845	KRT5	Keratin 5	12q12-q13
CAACTAATTCAATAAAA	990 (0.33)	3	Hs.436657	CLU	Clusterin	8p21-p12
TACCTGCAGAATAATAA	977 (0.33)	3	Hs.416073	S100A8	S100 calcium binding protein A8	1q21
GTTGTGGTTAATCTGGT	775 (0.26)	3	Hs.534255	B2M	Beta-2-microglobulin	15q21-q22.2
GTGGCCACGGCCACAGC	721 (0.24)	3	Hs.112405	S100A9	S100 calcium binding protein A9	1q21
GGAGTGTGCTCAGGAGT	717 (0.24)	3	Hs.504687	MYL9	Myosin, light chain 9, regulatory	20q11.23
ACTTTTTCAAAAAAAAA	648 (0.22)	3	Hs.349570	NCRNA00182	Non-protein coding RNA 182	Xq13.2
CACTACTCACCAGACGC	612 (0.20)	3	Hs.631491		Transcribed locus	
TACCATCAATAAAGTAC	605 (0.20)	3	Hs.544577	GAPDH	Glyceraldehyde-3-phosphate dehydrogenase	12p13
ACCCTTGGCCATAATAT	555 (0.19)	3	Hs.465808	HNRNPM	Heterogeneous nuclear ribonucleoprotein M	19p13.3-p13.2
TACATAATTACTAATCA	520 (0.17)	3	Hs.523789	NEAT1	Nuclear paraspeckle assembly transcript 1 (non-protein coding)	11q13.1
ACTAACACCCTTAATTC	491 (0.16)	3	Hs.631494	LOC100131754	Similar to NADH dehydrogenase subunit 2	1p36.33
GTAGGGGTAAAAGGAGG	451 (0.15)	3	Hs.631492		Transcribed locus	
TGCCTGCACCAGGAGAC	422 (0.14)	3	Hs.304682	CST3	Cystatin C	20p11.21
TAATAAAGAATTACTTT	407 (0.14)	3	Hs.654570	KRT15	Keratin 15	17q21.2
GAAATACAGTTGTTGGC	388 (0.13)	3	Hs.654447	CTSD	Cathepsin D	11p15.5
CAAGCATCCCCGTTCCA	375 (0.13)	2*	Hs.408470		Transcribed locus	
TACAGTATGTTCAAAGT	370 (0.12)	3	Hs.518525	GLUL	Glutamate-ammonia ligase (glutamine synthetase)	1q31
CATATCATTAAACAAAT	360 (0.12)	3	Hs.479808	IGFBP7	Insulin-like growth factor binding protein 7	4q12
ACACAGCAAGACGAGAA	343 (0.11)	3	Hs.721789		Transcribed locus	
ATTTGAGAAGCCTTCGC	336 (0.11)	3	Hs.703130		Transcribed locus	
CCACAGGAGAATTCGGG	325 (0.11)	3	Hs.201446	PERP	PERP, TP53 apoptosis effector	6q24
GTGCTGAATGGCTGAGG	323 (0.11)	3	Hs.632717	MYL6	Myosin, light chain 6, alkali, smooth muscle and non-muscle	12q13.2
GTGTGTTTGTAATAATA	296 (0.10)	3	Hs.369397	TGFBI	Transforming growth factor, beta-induced, 68 kDa	5q31
CAGGTTTCATATTCTTT	230 (0.08)	3	Hs.483444	CXCL14	Chemokine (C-X-C motif) ligand 14	5q31
ACGGAACAATAGGACTC	226 (0.08)	3	Hs.446429	PTGDS	Prostaglandin D2 synthase 21 kDa (brain)	9q34.2-q34.3
GATGCCGGCACAAAACT	223 (0.07)	3	Hs.146559	ANGPTL7	Angiopoietin-like 7	1p36.3-p36.2
CCCCCTGGATCAGGCCA	223 (0.07)	3	Hs.275243	S100A6	S100 calcium binding protein A6	1q21
GATGTGCACGATGGCAA	219 (0.07)	3	Hs.654380	KRT14	Keratin 14	17q12-q21
TAAGTAGCAAACAGGGC	214 (0.07)	3	Hs.643683	ITM2B	Integral membrane protein 2B	13q14.3
TCGAAGCCCCCATCGCT	211 (0.07)	3	Hs.631498	LOC100293090	Similar to DC24	
GTGACCTCCTTGGGGGT	210 (0.07)	3	Hs.433901	COX8A	Cytochrome c oxidase subunit 8A (ubiquitous)	11q12-q13
CTAGCCTCACGAAACTG	205 (0.07)	3	Hs.514581	ACTG1	Actin, gamma 1	17q25
CCCTGGGTTCTGCCCGC	205 (0.07)	3	Hs.433670	FTL	Ferritin, light polypeptide	19q13.33
GACCAGCTGGCCAAGAC	201 (0.07)	3	Hs.642660	C10orf116	Chromosome 10 open reading frame 116	10q23.2
GTTACCACAAGCCACAA	200 (0.07)	3	Hs.436037	MYOC	Myocilin, trabecular meshwork inducible glucocorticoid response	1q23-q24

†The percentage was based on the total number of tags in all three libraries (298834). * This 17 bp tag was not expressed in the library #625.

## Discussion

This is the first detailed SAGE gene expression profile reported for human TM tissue. Expression patterns in this study are consistent with the current understanding of normal trabecular meshwork physiology. Many expressed genes in the TM are related to extracellular matrix function, cell metabolism/defense/transport, cell signaling, and cell structure/adhesion [45]. As expected, genes involved in typical TM maintenance functions (including collagens, matrix metalloproteinases [MMPs], and tissue inhibitor of metalloproteinases [TIMPs]) are highly expressed, while those genes associated with stress or pathology are not highly expressed.

SAGE expression profiling of glaucomatous human TM would be a valuable complement to this study and could assist the exploration of disease-specific effects on tissue expression. TM tissue is available from POAG patients undergoing trabeculectomy surgery; however, surgical samples are small and yield insufficient RNA for SAGE analysis. Prospective enrollment of well documented glaucoma patients will be required to obtain tissue for such studies. Most patients with glaucoma have a history of medical or surgical treatment, which complicates interpretation of gene expression patterns.

Identifying candidate genes for POAG is a multifactorial and multistep process. Family-based linkage analysis has implicated more than fourteen loci, but only a few susceptibility genes have been identified [2]. The TM-specific gene expression data reported here contributes to the understanding of normal TM function, and constitutes a valuable resource to help prioritize and identify genes involved in the etiology of POAG.

## Appendix 1.

List of genes within known GLC1 loci that are expressed in all three HTM libraries. To access the data, click or select the words “Appendix 1.” This will initiate the download of a compressed (pdf) archive that contains the file.

## Appendix 2.

List of genes within known GLC1 loci that are expressed in at least one donor HTM library. To access the data, click or select the words “Appendix 2.” This will initiate the download of a compressed (pdf) archive that contains the file.
